# Supplementary material for: Urban Consumer Trust and Food Certifications in China
Source: Foods. 2020 Aug 21;9(9):1153. doi: 10.3390/foods9091153 (PMC7555469; doi:10.3390/foods9091153)
Supplement: Supplementary file 1 [file foods-09-01153-s001.pdf]

English version

QUESTIONARY – FOOD SAFETY AND FOOD QUALITY

PART 1 – BASIC INFORMATION

**AGE**	16-19	20-29	30-39	40-49	50-59	>60

**SEX**	MALE	FEMALE

**EDUCATION LEVEL**	HIGH SCHOOL	GRADUATE	POST GRADUATE

**MONTHLY INCOME**	<2000	2001-4000	4001-6000	6001-8000	8001-10000	>10001

**FAMILY STRUCTURE**	SINGLE / IN RELATIONSHIP	WIDOWED / SEPARATED	MARRIED NO CHILDREN	MARRIED WITH CHILDREN

PART 2 – EATING HABITS

FREQUENCY OF CONSUMPTION OF THE FOLLOWING FOOD CATEGORIES

	DAILY	3 TIMES A WEEK	WEEKLY	MONTHLY	RARELY/ NEVER
STAPLE FOOD (rice,flour and derivates)					
MILK AND DERIVATES (cheese,yogurt,etc.)					
EGGS					
MEAT					
FISH					
VEGETABLES					
FRUIT					

WHICH OF THE FOLLOWING CHARACTERISTICS ARE IMPORTANT IN CHOOSING FOOD? (min 1; max 3)

TASTE ☐ SMELL ☐ PACKAGE ☐ PRICE ☐

INGREDIENTS ☐ EXPIRATION DATE ☐ BRAND ☐ COLOUR ☐

HOW OFTEN DO YOU BUY FOOD?

DAILY ☐ > 3 TIMES A WEEK ☐

< 3 TIMES A WEEK ☐ < 1 TIMES A WEEK ☐

WHERE DO YOU USUALLY BUY FOOD?

SUPERMARKET ☐ LOCAL MARKET ☐ LITTLE RETAILERS ☐

WHY DO YOU CHOOSE THAT PLACE? (min 1; max 2)

POSITION ☐ LOW PRICES ☐ RELIABILITY ☐

BRAND OF THE MARKET ☐ PRODUCT'S QUALITY ☐

HOW OFTEN DO YOU READ THE LIST OF INGREDIENTS OF THE PRODUCTS THAT YOU BUY? (FROM 1 TO 5: 1 = NEVER; 5 = ALWAYS)

1<br>NEVER	2<br>A FEW TIMES	3<br>SOMETIMES	4<br>MOST OF THE TIMES	5<br>ALWAYS
☐	☐	☐	☐	☐

WHAT IS THE MEANING OF THIS MARK FOR YOU?

生产许可

BETTER QUALITY ☐ SAFER ☐

MORE TRUSTABLE ☐ BETTER TASTE ☐

DON'T KNOW ☐

DO YOU USUALLY CHECK IF THIS MARK IS PRINTED ON THE LABELS OF THE PRODUCTS THAT YOU BUY?

生产许可

YES ☐

NO ☐

PART 3 – FOOD SAFETY

HOW MUCH CONFIDENCE YOU HAVE IN THE FOOD THAT YOU EAT?
(FROM 1 TO 5: 1 = NO CONFIDENT; 5 = TOTALLY CONFIDENT)

1 NOT CONFIDENT	2 LOW CONFIDENCE	3 MEDIUM CONFIDENCE	4 HIGH CONFIDENCE	5 TOTALLY CONFIDENT
☐	☐	☐	☐	☐

HOW MUCH CONFIDENCE YOU HAVE IN THE SAFETY OF THE FOLLOWING FOOD CATEGORIES?
(FROM 1 TO 5: 1 = NO CONFIDENT; 5 = TOTALLY CONFIDENT)

	1 NOT CONFIDENT	2 LOW CONFIDENCE	3 MEDIUM CONFIDENCE	4 HIGH COFIDENCE	5 TOTALLY CONFIDENT
STAPLE FOOD					
MILK AND DERIVATES					
EGGS					
MEAT					
FISH					
VEGETABLES					
FRUIT					

HAVE YOU EVER HAD EXPERIENCE WITH UNSAFETY FOOD?

YES ☐ NO ☐

IF YES: WITH WHICH ONE OF THE FOLLOWING FOOD CATEGORIES?

STAPLE FOOD ☐ MILK AND DERIVATES ☐ MEAT ☐

FISH ☐ VEGETABLES ☐ FRUIT ☐

EGGS ☐

WHICH ONE OF THE FOLLOWING FOOD CATEGORIES YOU RETAIN THE LESS SAFE?

STAPLE FOOD	☐	MILK AND DERIVATES	☐	MEAT	☐
FISH	☐	VEGETABLES	☐	FRUIT	☐
EGGS	☐				

IN YOUR OPINION, WHICH OF THE FOLLOWING CHARACTERISTICS ARE RELATED TO FOOD SAFETY? (min 1; max 3)

TASTE	☐	SMELL	☐	PACKAGE	☐	PRICE	☐
INGREDIENTS	☐	EXPIRATION DATE	☐	BRAND	☐	COLOUR	☐

HOW CONCERNED ARE YOU ABOUT EACH OF THE FOLLOWING ISSUES RELATED TO THE FOOD SAFETY?
(FROM 1 TO 5: 1 = NOT AT ALL; 5 = VERY CONCERNED)

	1 NOT AT ALL	2	3 NORMALLY CONCERNED	4	5 VERY CONCERNED
OVERUSE OF ADDITIVES					
RESIDUES OF HORMONES/ ANTIBIOTICS					
ANIMAL DISEASES					
CONTAMINATION (BACTERIA AND VIRUS)					
RESIDUE OF PESTICIDES AND FERTILIZER					
OGM					

HOW CONCERNED ARE YOU ABOUT THE LAWS OF YOUR COUNTRY RELATED TO FOOD SAFETY?
(FROM 1 TO 5: 1 = NOT AT ALL; 5 = VERY CONCERNED)

1 NOT AT ALL	2	3 NORMALLY CONCERNED	4	5 VERY CONCERNED
☐	☐	☐	☐	☐

WHICH OF THE FOLLOWING CERTIFICATIONS DO YOU KNOW?

ISO9000 质量认证	☐	无公害食品认证	☐
绿色食品认证	☐	有机食品认证	☐
食品可追溯认证	☐	I don't know any	☐

HOW MUCH DO YOU AGREE WITH THE FOLLOWING SENTENCES
(from 1 to 5: 1= Do not agree; 5= I totally agree)

	1	2	3	4	5
Chinese governament is interested in food safety					
Chinese governament provides informations about food safety					
Chinese governament's laws about food safety are strict enough					
Today the food is safer than 10 years ago					
The authorities retain consumer's health more important than the profit of companies					
The opinions of the scientific community about food safety are not related to political and commercial interests.					

HOW MUCH DO YOU THINK THAT THE PRICE OF A PRODUCT IS RELATED TO ITS SAFETY? (FROM 1 TO 5: 1= 0% RELATION; 5= 100% RELATION)

1<br>0%<br>RELATED	2<br>LOW<br>RELATED	3<br>50%<br>RELATED	4<br>HIGH<br>RELATED	5<br>100%<br>RELATED
☐	☐	☐	☐	☐

HOW MUCH ARE YOU WILLING TO PAY MORE THAN THE STANDARD PRICES, TO BUY A SAFER PRODUCT?

	**0%**	**10%**	**25%**	**50%**	**DOUBLE PRICE**
STAPLE FOOD					
MILK AND DERIVATES					
EGGS					
MEAT					
FISH					
VEGETABLES					
FRUIT					

PART 4 – FOOD QUALITY

IN YOUR OPINION, WHAT IS THE QUALITY LEVEL OF THE FOOD THAT YOU BUY? (FROM 1 TO 5: 1= LOW QUALITY; 5= HIGH QUALITY)

1 VERY LOW QUALITY	2 LOW QUALITY	3 MEDIUM QUALITY	4 HIGH QUALITY	5 VERY HIGH QUALITY
☐	☐	☐	☐	☐

IN YOUR OPINION, WHICH OF THE FOLLOWING CHARACTERISTICS ARE MORE IMPORTANT TO DEFINE FOOD QUALITY? (min 1; max 3)

TASTE	☐	SMELL	☐	PACKAGE	☐	PRICE	☐
INGREDIENTS	☐	EXPIRATION DATE	☐	BRAND	☐	COLOUR	☐

WHAT IS THE MOST IMPORTANT THING FOR YOU, WHEN YOU HAVE TO CHOOSE BETWEEN 2 OR MORE PRODUCTS OF THE SAME CATEGORY?

PRICE ☐ BRAND ☐ APPEARENCE ☐

IN YOUR OPINION, WHAT IS THE MAIN FACTOR THAT INFLUENCE FOOD QUALITY? (min 1; max 2)

INGREDIENTS'S ORIGIN	☐	MANIPULATION	☐
INGREDIENT'S QUALITY	☐	BRAND	☐

IN YOUR OPINION, HOW MUCH IMPORTANT IS THE FOOD QUALITY IN THE FOLLOWING FOOD CATEGORIES? (FROM 1 TO 5: 1= NOT IMPORTANT; 5= VERY IMPORTANT)

	1 NOT IMPORTANT	2	3 MEDIUM IMPORTANT	4	5 VERY IMPORTANT
STAPLE FOOD					
MILK AND DERIVATES					
EGGS					
MEAT					
FISH					
VEGETABLES					
FRUIT					

HOW MUCH DO YOU THINK THAT THE PRICE OF A PRODUCT IS RELATED TO ITS QUALITY? (FROM 1 TO 5: 1= 0% RELATION; 5= 100% RELATION)

1 0% RELATED	2 LOW RELATED	3 50% RELATED	4 HIGH RELATED	5 100% RELATED
☐	☐	☐	☐	☐

HOW OFTEN DO YOU BUY A PRODUCT WITH AN HIGHER PRICE BECAUSE OF ITS QUALITY? (FROM 1 TO 5: 1= NEVER; 5= ALWAYS)

1 NEVER	2 A FEW TIMES	3 SOMETIMES	4 MOST OF THE TIMES	5 ALWAYS
☐	☐	☐	☐	☐

HOW MUCH ARE YOU WILLING TO PAY MORE THAN THE STANDARD PRICES, TO BUY A PRODUCT WITH AN HIGHER QUALITY?

	0%	10%	25%	50%	DOUBLE PRICE
STAPLE FOOD					
MILK AND DERIVATES					
EGGS					
MEAT					
FISH					
VEGETABLES					
FRUIT					

HOW MUCH ARE YOU WILLING TO PAY MORE THAN THE STANDARD PRICES, TO BUY A PRODUCT WITH THOSE MARKS PRINTED ON THE LABELS?

	0%	10%	25%	50%	DOUBLE PRICE
**GREEN FOOD**					
**ORGANIC FOOD**					

DO YOU THINK THAT THE QUALITY OF IMPORTED PRODUCT IS HIGHER THAN CHINESE PRODUCT?

YES ☐ NO ☐

HOW MUCH ARE YOU WILLING TO PAY MORE THAN THE STANDARD PRICES TO BUY FOOD IMPORTED FROM ANOTHER COUNTRY BECAUSE OF ITS QUALITY?

0% ☐ 10% ☐ 25% ☐

50% ☐ DOUBLE PRICE ☐
